# Injective Therapies for Managing Sacroiliac Joint Pain in Spondyloarthropathy: A Systematic Review and Meta-Analysis

**DOI:** 10.3390/jcm14041294

**Published:** 2025-02-15

**Authors:** Tosca Cerasoli, Giuseppe Filardo, Antongiulio Favero, Vito Gaetano Rinaldi, Laura Di Maio, Giulio Maria Marcheggiani Muccioli, Stefano Zaffagnini

**Affiliations:** 1 II Orthopaedic and Traumatologic Clinic, IRCCS Istituto Ortopedico Rizzoli, 40136 Bologna, Italy; antongiulio.favero@ior.it (A.F.); vitogaetano.rinaldi@ior.it (V.G.R.);; 2 Faculty of Biomedical Sciences, Università della Svizzera Italiana, 6900 Lugano, Switzerland; 3 Clinical Trial Center, IRCCS Istituto Ortopedico Rizzoli, 40136 Bologna, Italy

**Keywords:** sacroiliac joint, sacroiliac joint injections, sacroiliac joint pain, spondyloarthropathy, injections in spondyloarthropathy, steroids in spondyloarthropathy

## Abstract

**Background**: The most effective treatment approach for sacroiliac joint (SIJ) pain in spondyloarthropathy (SpA) patients remains unclear. This systematic review and meta-analysis aimed to assess the safety and effectiveness of different injective therapies for SIJ pain in SpA patients. **Methods**: A comprehensive literature search was conducted up to January 2024. The inclusion criteria encompassed studies in English, including comparative and non-comparative studies, and case series. A meta-analysis was performed on the available data. The “Checklist for Measuring Quality” by Downs and Black was used to evaluate the quality of included papers. **Results**: A total of 17 studies involving 494 patients were included: 12 prospective case series, 1 retrospective comparative study, 2 prospective comparative studies, and 2 randomized controlled trials. Steroid injections were analyzed in 15 studies, etanercept in 1, and infliximab in 1. A meta-analysis of 375 patients receiving steroid injections showed a significant reduction in visual analog scale (VAS) scores from 8.2 pre-treatment to 3.2 (*p* < 0.001) at short-term follow-up, with stability at mid-term follow-up (VAS 3.3, *p* < 0.001) and worsening at the last follow-up (VAS 5.1, *p* < 0.001). The failure rate was 13% (*p* = 0.019), and one study reported a 12.5% complication rate. Biologic therapies showed no complications or failures, with improvements in both VAS and BASDAI scores. **Conclusions**: Intra-articular steroid injections are effective and safe for SIJ pain in SpA patients, although their efficacy diminishes over time, and not all patients respond to treatment. Biologic therapies have shown promising results, but further research is needed to confirm their long-term efficacy.

## 1. Introduction

Spondyloarthropathies (SpAs) are a group of chronic inflammatory rheumatic musculoskeletal diseases characterized by the absence of a rheumatoid factor and a predilection for the axial skeleton [1]. They include axial spondyloarthritis (AS), juvenile spondylitis, psoriatic arthritis, reactive arthritis, and enteropathic arthritis (associated with Chron’s disease and ulcerative colitis) [1]. Among this broad spectrum of pathologies, the most common manifestations are sacroiliitis and spondylitis, presenting clinically with back pain and sacroiliac joint (SIJ) pain [2]. Sacroiliitis is often one of the first clinical symptoms in AS and juvenile spondylitis, and even without radiological evidence of SIJ damage, it constitutes a significant diagnostic element as well as a major burden for patients [3]. Over the years, various treatment options have been proposed to manage SIJ pain related to SpA. Non-steroidal anti-inflammatory drugs (NSAIDs) and physical therapy are the first treatment line, but have shown limited long-term efficacy. The use of disease-modifying anti-rheumatic drugs (DMARDs) like sulfasalazine, methotrexate, and leflunomide remains widespread, although they provide limited benefit for patients experiencing low back pain, and they are not recommended in patients affected by a purely axial disease [4]. In recent years, biological agents, such as tumor necrosis factor (TNF-α) or IL-17 antagonists, have been proposed in patients with specific characteristics associated with a higher likelihood of response: elevated C-reactive protein levels (CRP), the presence of inflammation on MRI-SIJ, and the presence of radiographic sacroiliitis. However, SIJ pain is not always related to pathological imaging, nor to a high level of CRP. Moreover, the literature highlights the high costs and associated risks of infection with these long-term treatments. Likewise, the long-term use of glucocorticoids as oral medication is also discouraged. On the other hand, local injections of glucocorticoids at the site of musculoskeletal inflammation should be considered according to the most recent recommendations of ASAS-EULAR, despite some controversial evidence, and other injective procedures have been proposed to address SIJ pain [4]. Despite the widespread use of steroid injections for SIJ pain relief, there is a lack of long-term comparative data evaluating their effectiveness against biologic therapies. Moreover, current evidence does not adequately differentiate responses based on specific SpA subtypes, limiting the ability to tailor treatment recommendations to individual patient characteristics.

The aim of this systemic review and meta-analysis was to analyze the safety and effectiveness of the different SIJ injective options to provide evidence-based results to guide physicians in the management of SpA.

## 2. Materials and Methods

Following the guidelines of the Reporting Items for Systematic Reviews and Meta-Analyses (PRISMA) statement (www.prisma-statement.org accessed on 1 January 2024), a review protocol was prepared. Two authors independently conducted a literature search on PubMed, Scopus, and Embase from inception until 17 January 2024, using the search string (“sacroiliac” OR “sacro-iliac”) AND (“inject” OR “conservative”). The authors applied no filters to the search. Exclusion criteria were languages different from English, case reports or case series with less than 5 patients enrolled, preclinical studies, cadaveric studies, studies involving children, or patients not affected by SpA. Efficacy and safety of different injection therapies were quantified. All studies meeting the inclusion criteria were reported as detailed in Table 1. A meta-analysis was conducted when at least three studies were present on the same treatment, to quantify the reported data on injective therapies.

### 2.1. Data Extraction

Two reviewers (T.C. and A.F.) independently screened the articles by title, abstract, and full texts, and a third reviewer (V.G.R.) contributed to the screening process when a consensus was not reached. Using Excel (Microsoft, Redmond, WA, USA), a data extraction form was created, reporting the following: authors, titles, year of publication, PMID, journal of publication, type of study, blindness, number of treated patients, number of patients at final follow-up, number of SIJs treated, age, sex, symptoms, previous treatments, type and details of the treatment, follow-up, scores, failures, complications, and study conclusions.

### 2.2. Assessment of Risk of Bias and Quality of Evidence

The risk of bias and the quality of the papers were assessed by the two reviewers using the “Checklist for Measuring Quality” by Downs and Black [20], with the third reviewer contributing to the screening process when a consensus was not reached. The checklist consists of 27 ‘yes’ or ‘no’ questions in 5 sections (overall study quality, ability to infer conclusions of the study, study bias, selection bias, and power of the study).

### 2.3. Statistical Analysis

All statistical analyses were conducted with Jamovi software (The Jamovi Project—Jamovi Version 1.6 2021). To calculate the odds ratios and statistical parameters (95% confidence interval, regression slopes and intercepts), random-effects models were used (using restricted maximum likelihood estimators). The publication bias was assessed via regression tests for funnel plot asymmetry using sample size, standard error, and sample variance as predictors. Forest plots were used to represent results (*p* value < 0.05 was considered significant). Inconsistency statistics (I2 > 75% was considered as high) were used as a heterogeneity assessment.

## 3. Results

Study selection

**Figure 1:**
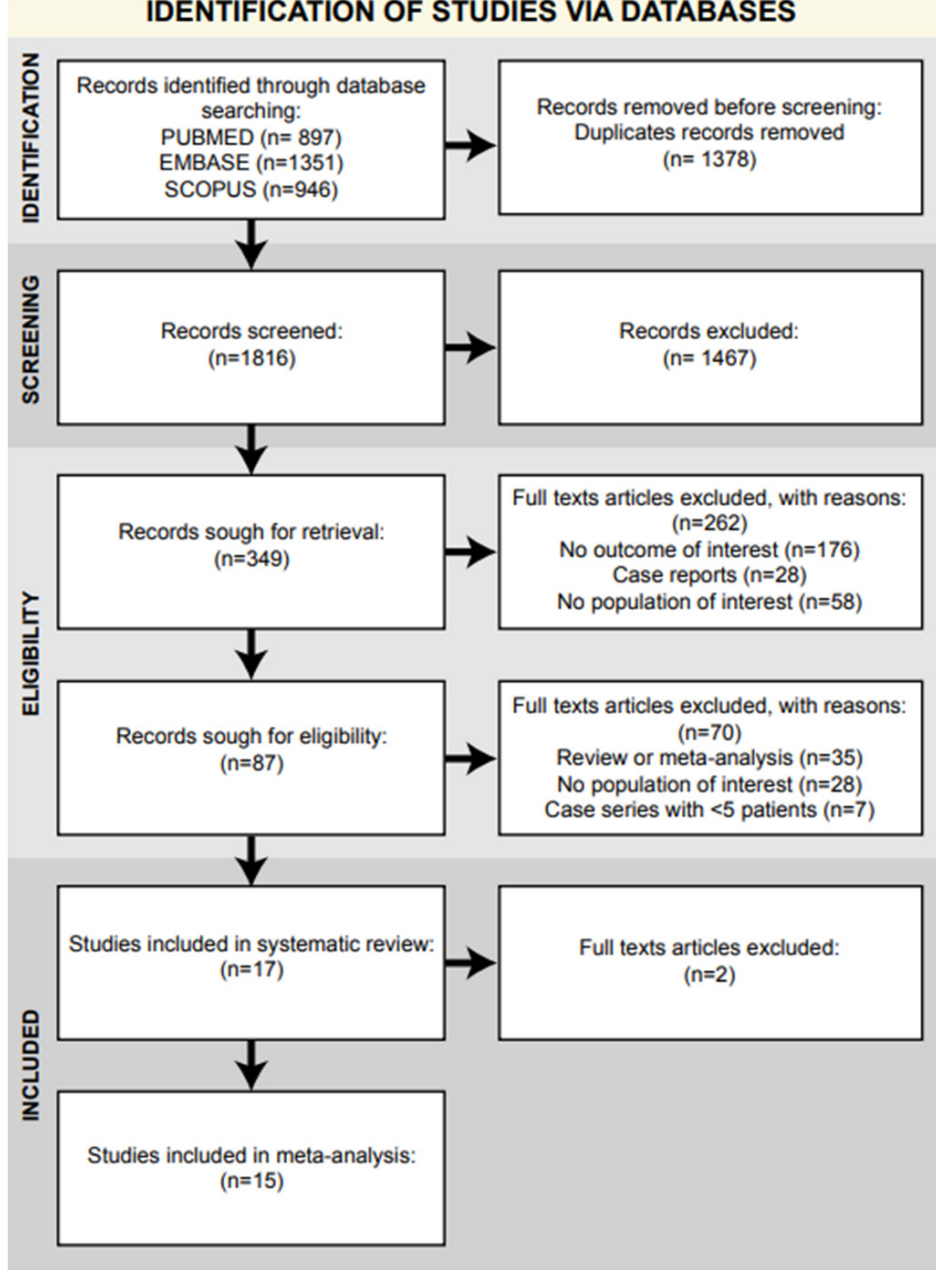
PRISMA flow chart.

A total of 3194 articles were identified using the search string (“sacroiliac” OR “sacro-iliac”) AND (“inject*” OR “conservative”) in the databases: 897 in PubMed, 1351 in Embase, and 946 in Scopus. After the removal of duplicates, 1816 articles were screened by title and abstract, and 87 articles were assessed for eligibility and obtained in their full-text versions. To ensure wider data accessibility for all authors and readers, only articles with an available full text in English were included. After full-text evaluation, 17 articles were included in the systematic review: 12 prospective case series, 1 retrospective comparative study, 2 prospective comparative studies, and 2 randomized controlled trials (1 double blind). The entire selection process is reported in the PRISMA flow chart (Figure 1).

The effect of steroid injections was examined in 15 studies, which were included in the meta-analysis. The steroids chosen for injection were triamcinolone acetate (TAC), in nine studies, and methylprednisolone, betamethasone, and cortivazol, each in two studies. The other two articles were prospective case series describing the effect of intra-articular injections of etanercept and infliximab.

**Figure 2:**
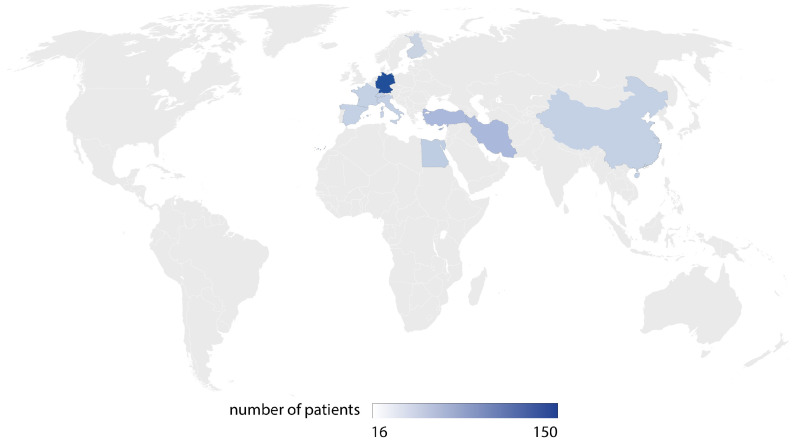
Geographical distribution of included studies.

The majority of the studies were conducted in Germany (six studies), in France, and in Finland (two studies each). The systematic review included 494 patients, and their geographical distribution is shown in Figure 2.

Male patients constitute 58% of the population included, and 30% were female (in 12% the patients’ genders were not specified). The distribution of studies per year shows that SIJ steroid injections remain a topic of interest, with three studies published in 2022 alone.

In one of the studies included, the SIJ injection was performed using only anatomical landmarks [8]; in all the other studies, radiological guidance was used. Fluoroscopy was the most used approach (six studies), ultrasonography (US) guidance was reported in four articles (three of them published in 2022), computer axial tomography (CT) in four studies, and magnetic resonance imaging (MRI) in two studies. A total of 15 studies reported an intra-articular injection approach; 1 study using a peri-articular approach tested the peri-articular effect of methylprednisolone acetate compared to placebo injection [13], and 1 study compared the effect of peri-articular and intra-articular SIJ injections of triamcinolone [14].

Six studies reported an injection volume of corticosteroids of 1 mL, four studies of 3 mL, two of 2 mL, two of 1.5 mL, and in one study the volume was not reported. Infliximab and etanercept injections were administered both at a volume of 2 mL. One study reported only an early follow-up (<1 month), nine studies a mid-term follow-up (up to 3 months), and five studies a last follow-up of minimum 6 months.

The included studies showed considerable heterogeneity in steroid type (triamcinolone acetate, methylprednisolone, betamethasone, cortivazol), injection volume (ranging from 1 mL to 3 mL), and follow-up duration. These factors likely contributed to the variability in the reported outcomes and should be considered when interpreting the results.

### 3.1. Systematic Review Results

Out of the 494 patients included, 375 received steroid injections (185 males, 145 females among the studies that reported sex rate), 37 male patients were treated with infliximab, 16 patients (sex rate not reported) with etanercept, and 66 patients (33 males, 14 females, when reported) were part of control groups treated with placebo injections or oral therapy. All 17 studies documented both failures and complications. The complications and failures of the steroid groups are reported and analyzed in the following paragraph on the meta-analysis results.

In the studies testing biological therapies, the rates of complications and failures were zero. Both studies reported visual analog scale (VAS) scores and Bath Ankylosing Spondylitis Disease Activity Index (BASDAI) scores at a last follow-up at 3 months. The improvement in BASDAI scores ranged from 2.7 for patients treated with etanercept to 3.3 points for patients treated with infliximab; similarly, the improvement in VAS score was around 2 points for patients treated with infliximab [19] and 4 points for patients treated with etanercept [9].

Among the comparative studies, two compared steroid injections with oral therapy and reported both BASDAI and VAS scores. In the group treated with TAC SIJ injection, an improvement in the VAS score from 7.9 to 2.9 was observed at the mid-term follow-up (3 months), with a slightly worsening score at the last follow-up (3.2 at 6 months) [12]. Similarly, the group treated with methylprednisolone injection showed a comparable improvement of 6.3 points in their VAS scores at the mid-term follow-up (2 months), but no further follow-up data were recorded [1]. In the control groups, patients treated with oral indomethacin exhibited an improvement in VAS scores from 8 to 4.8 at the last follow up [12], while patients treated with a combination of oral steroids, NSAIDs, and sulfasalazine showed an improvement of 0.3 points in their VAS scores [1]. Regarding BASDAI scores, patients injected with TAC demonstrated a consistent improvement of 1.4 points [12] which persisted at the mid-term and last follow-ups (6 months). On the other hand, those treated with methylprednisolone showed an improvement of 5.6 points [1] which remained stable at 1 months and 2 months of follow-up. In the control groups, patients treated with oral indomethacin experienced a worsening in their BASDAI scores at the final follow-up [12], while the group treated with a combination of perioral steroids, NSAIDs, and sulfasalazine showed no improvement in their BASDAI scores [1].

Two studies compared steroid injections to saline injections. Patients treated with peri-articular methylprednisolone injection exhibited an improvement of 1.7 points in their VAS scores at the last follow-up (2 months) [13]. Conversely, those receiving intra-articular cortivazol injections demonstrated a more substantial improvement of 5.5 points at the last follow-up (1 month) [15]. Among the control groups, one study reported an improvement of approximately 2 points at the last follow-up for patients treated with 1.5 mL of intra-articular saline solution [15]. However, no improvement was observed in patients treated with 3 mL of peri-articular saline injection at their last follow-up [13].

Among patients included in all control groups, the failure rate was 26%. The highest failure rates were recorded in the control group that received as placebo a peri-articular saline injection [13] and the group treated with peri-articular steroid injection [14], with no improvements observed.

### 3.2. Meta-Analysis Results

**Figure 3:**
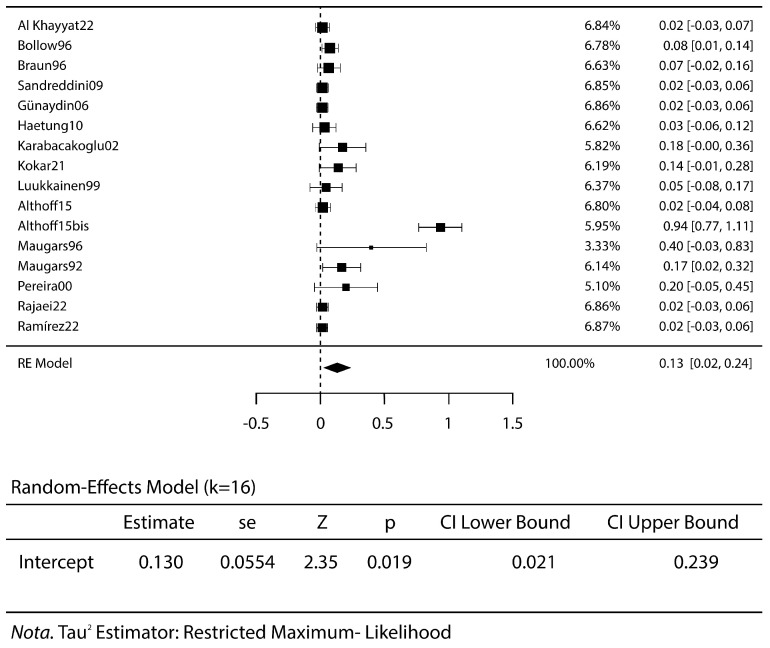
Failure rate of steroid injective therapy: forest plot and proportions [1,4,5,6,7,8,10,11,12,13,14,15,16,17,18].

This pooled analysis included 382 patients treated with steroid injections. The mean age was 35.8 years. The failure rate was 13% (*p* < 0.019) (Figure 3).

**Figure 4:**
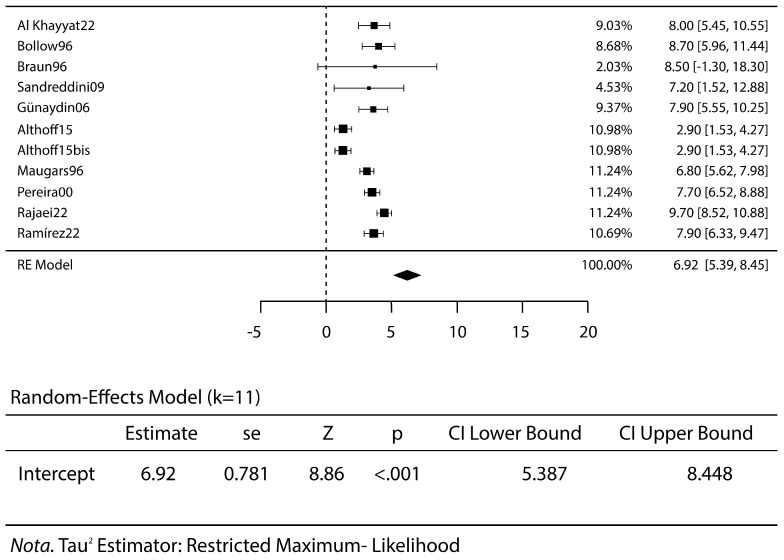
VAS scores before steroid injective treatment: forest plot and proportions [1,4,5,6,7,8,14,15,17,18].

**Figure 5:**
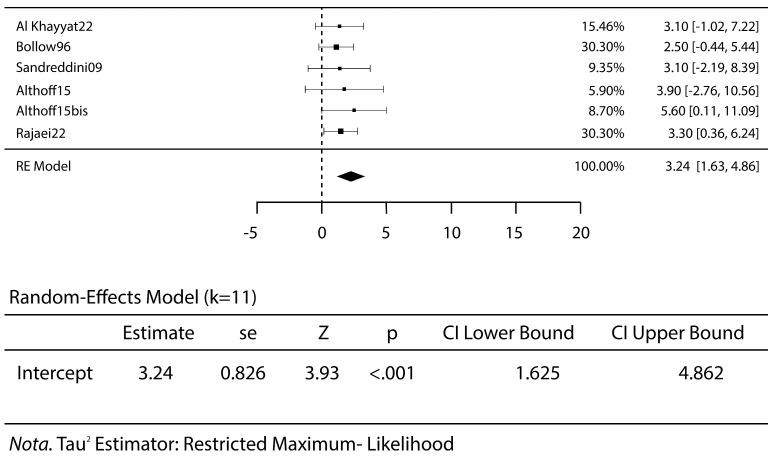
The VAS scores at short-term follow-up: forest plot and proportions. The decreases in the VAS scores highlight the reductions in the patients’ pain [1,5,6,8,14].

**Figure 6:**
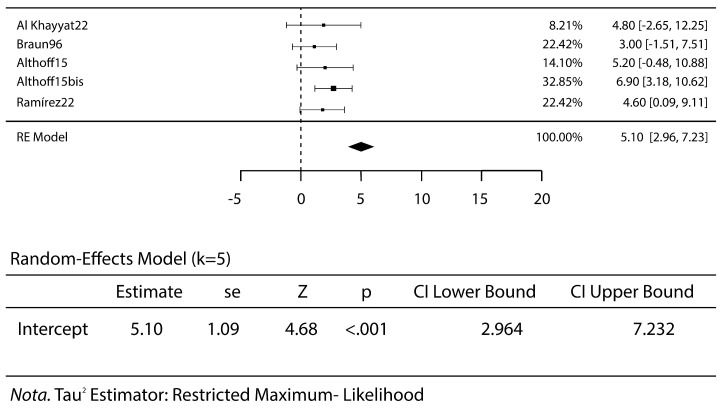
The VAS scores at short-term follow-up: forest plot and proportions. The slight increases in the VAS levels indicate how the therapy’s effect diminishes over time [5,7,14,18].

All studies reported no complications, except for one that reported three complications for a total rate of 12.5%: one case of transient anesthesia of the perineum and two cases of acute transient pain the first night after undergoing the procedure [15]. The VAS scores were meta-analyzed before treatment, at early follow-up, at mid-term follow-up and at the last follow-up. A high VAS score relates to a severe pain for the patients. The mean VAS score before treatment was 7.0 (Figure 4), VAS at the early follow-up was 3.2 (*p* < 0.001) (Figure 5) and remained stable at mid-term follow-up (VAS 3.3, *p* < 0.001), with a decrease in pain and discomfort for the patients. A worsening was reported at the last follow-up, with a VAS score of 5.1 (*p* < 0.001) (Figure 6).

A comparative evaluation of steroid versus biologic injections showed that both treatments provided significant pain relief, as indicated by reductions in the VAS and BASDAI scores. However, while steroid injections demonstrated short-term efficacy, biologic injections appeared to maintain more stable improvements over time. The limited number of studies investigating biologic therapies prevents definitive conclusions, but their zero failure rate and absence of reported complications suggest a potentially valuable alternative.

### 3.3. Risk of Bias and Quality of Evidence

**Figure 7:**
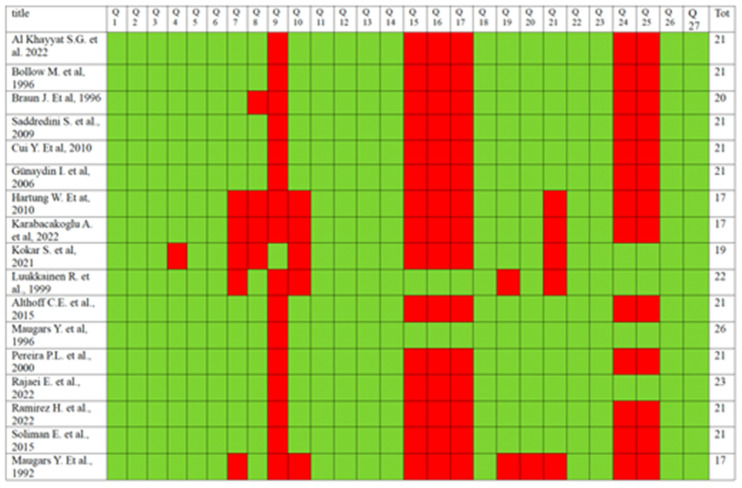
Downs and Black’s checklist, green indicates a positive score (criteria met), while red indicates a negative score (criteria not met) [1,4,5,6,7,8,9,10,11,12,13,14,15,16,17,18,19].

Downs and Black’s checklist for assessing the risk of bias gives each study an excellent ranking for scores ≥26, a good ranking for scores from 20 to 25, fair for scores between 15 and 19, and poor for scores ≤14 points [20]. According to these criteria, 1 study was classified as excellent, 12 as good, and 4 as fair. No studies were classified as poor. Among the studies meta-analyzed, one was classified as excellent, eight as good, and four as fair. The quality of the studies included was lowered by the paucity of randomized control trials and double-blind studies, as well as the lack of probability values (Figure 7).

## 4. Discussion

The primary finding of this systematic review and meta-analysis is that corticosteroids are the most documented injective approach for relieving SIJ pain in patients with SpA, showing an overall significant effectiveness. However, while corticosteroid injections have shown to be generally safe, not all patients respond to treatment, and a significant reduction in pain relief has been documented over time.

Steroid injections have been utilized in patients with SpA and axial pain since 1996, particularly SIJ pain [6], relying on their anti-inflammatory properties and pleiotropic effects, which involve downregulating pro-inflammatory cytokines and chemokines while upregulating the production of anti-inflammatory genes [21]. Although systemic glucocorticoids have been used in the management of SpA in the past, the evidence supporting their effectiveness in reducing SIJ pain is limited [3,4,6,8,9,12,13,14,15,16,19,20,21,22,23], and the literature has extensively documented the well-known side effects associated with systemic glucocorticoids. For these reasons, systemic steroids are not recommended as preferred treatment options [3,4,6,8,9,12,13,14,15,16,19,20,21,22,24]. On the other hand, as documented by this systematic review, the injective approach is widely used, and is still the subject of scientific investigation to understand its potential effects in the affected joints. Intra-articular steroid injections have been proven to be effective, and this meta-analysis also underlines the safety of the procedure, with only a few minor complications reported.

Targeting the affected joint with steroid therapy allows physicians to enhance the anti-inflammatory effects while reducing the risks of systemic complications [25]. To this end, radiological guidance is recommended for the accurate administration of steroid injections into the SIJ. Literature reports indicate that blind injection accurately reaches the intra-articular space in only 22% of patients [26], which is in line with the administration chosen in the analyzed studies. Among the studies included in this systemic review and meta-analysis, only one reported the choice of administrating the injection without any radiological guidance [8]. A blind injection may result in a peri-articular steroid injection. The peri-articular approach has been investigated in two comparative studies. The study conducted in 1999 by Luukkainen et al. compared the efficacy of peri-articular steroid injections to a placebo injection [13], reporting early promising results. However, the study conducted by Althoff et al. in 2015 comparing the efficacy of intra- and peri-articular steroid injections [14] highlighted the advantages of the intra-articular approach. This study assessed the VAS score at 3 months and 6 months of follow-up. The findings revealed a consistent difference of 2 points of reduction in VAS scores between the peri-articular and intra-articular approaches, providing evidence supporting the conclusion that the intra-articular approach is more effective [14]. The literature also suggests a combined approach for steroid injections in SIJ pain [27]. However, it is important to note that these recommendations are based on studies not specific to SpA, while, based on the results of this systematic SpA-focused literature analysis, the most supported indication is the intra-articular guided approach. Nevertheless, this remains a limitation of the present study, as the lack of direct comparisons prevents a definitive assessment of the best imaging guidance technique.

A consensus on the most suitable guidance to administer injections has not been reached, yet. In recent years, there has been growing interest in the use of ultrasound (US) guidance as a safer technique that avoids radiation exposure for both patients and physicians. US guidance offers advantages such as the improved visualization of nerves and vessels, and it can be performed in a doctor’s office and repeated as necessary [26]. In fact, all the three studies published in 2022 reported the use of US guidance [1,5,18]. Nevertheless, US is not the only method reported, and previous authors documented the results of injections guided either by fluoroscopy or MRI. However, injections under MRI guidance are still rarely performed, and MRI evaluation is commonly executed to assess the reduction in bone edema or in contrast enhancement before and after the steroid injection. The majority of the analyzed studies reported a consistent reduction in the parameters describing disease activity, such as bone edema or contrast enhancement [4,6,7,17,28].

The effectiveness of the injective therapy was mainly assessed through clinical scores, with the VAS being the most utilized tool to investigate pain symptoms. The included studies reported the scores at different follow-up time points, which allowed for an in-depth analysis of the results. In fact, while the recent literature suggested an average duration of improvement of eight months [25], this meta-analysis underlined a lower benefit of corticosteroids over time. A one-month follow-up is considered a short-term evaluation period. During this temporal range, the reduction in VAS score was approximately 5 points, indicating a decrease in SIJ pain. This pain reduction was maintained for up to 3 months following the injection. However, beyond this timeframe, the efficacy of steroid injections tended to decline. At a follow-up of 6 months or longer, the VAS score was found to be 3 points lower than the initial value. The meta-analysis highlighted the short-term efficacy of steroid injection, which may suggest that repeated injection cycles over time could be beneficial, particularly considering the progressive nature of SpA. However, multiple steroid injections can increase the risk of inducing local chondrolysis and osteoporosis, the risk of septic arthritis, and can increase the post-operative pain scores in the case of surgery [29,30,31,32].

Functional limitations are another important symptom presented by SpA, which were more specifically explored by BASDAI scores [1,2,3,4,8,9,12,13,14,19]. Improvement in the BASDAI is a significant clinical outcome as it directly represents the limitations on patients’ daily routine. The BASDAI is a functional score that more faithfully represents the difficulties encountered in the daily activities. This score was also used to compare the effects of different corticosteroids. A higher BASDAI score suggests fewer or no limitations in daily activities. Rajae et al. achieved the best results with methylprednisolone injections [1], while patients treated with TAC by Kokar et al. experienced a smaller reduction in BASDAI score [12]. Yet, these comparative results are based on a few studies with contradictory findings, as confirmed by the study of Krishnan et al. showing that no differences in the efficacy of the chosen steroids have been demonstrated [33]. Also, with regard to pain reduction, the results are inconclusive. While an overall improvement has been documented in the short term versus placebo, heterogeneous results were documented in terms of VAS reduction when comparing different corticosteroids. Intra-articular cortivazol showed better results [15], but due to the different injection approach and the small sample size it is not possible to conclude the overall superiority of a specific steroid. Moreover, a meaningful comparison is impaired by the differences in terms of injection approaches. Finally, it is important to underline that placebo still plays an important part in the clinical outcome document. As shown for other injective treatments [34], the placebo effect is significant both in terms of functional improvements and pain perception. The high, long-lasting, and heterogeneous effects on the scales commonly used in clinical trials further highlight that the impact of placebo should not be overlooked in the research on injective approaches.

The management of SpA has seen the emergence of new medications, which have been proposed in recent years when traditional treatment fails for specific indications. Anti-tumor necrosis factor (TNF) agents can also be administered systemically to address SIJ pain, but their use is recommended only under specific conditions, such as persistent high disease activity, elevated CRP levels, or positive imaging for sacroiliitis [3]. Some studies have also reported the local use of anti-TNF agents. Infliximab, a monoclonal antibody targeting TNF-alpha, was first reported in a case study in 2009 for HLA B27-negative patients with sacroiliitis, showing promising results after a 2-year follow-up. Subsequently, its use was tested by Soliman et al. in 2015, involving 37 patients, with promising outcomes [19]. Another anti-TNF agent, etanercept, which acts as a monoclonal antibody against the soluble receptor for TNF-alpha, was evaluated as a local therapy in 16 patients by Cui et al. Their patients received three injections, one at the first month and one after two months [9]. The reductions in VAS and BASDAI scores were greater than those achieved with infliximab. However, both studies provided short-term follow-up, making it impossible to compare the results to the long-term follow-up of the steroid injections. While these results are promising, the lack of case series and trials directly comparing biologic therapies with steroid injections diminishes their significance. Moreover, the current recommendations for anti-TNF therapy are primarily formulated for subcutaneous or intravenous administration [3], and the small number of available studies prevents definitive conclusions regarding their efficacy and safety. Cost considerations may also play a significant role in treatment selection. Steroid injections represent a cost-effective option for short-term pain relief, but their diminishing effectiveness over time may necessitate repeated procedures, potentially increasing long-term healthcare costs. In contrast, biologic therapies, while more expensive upfront, may reduce the need for recurrent interventions and prolonged systemic medication use. Given these aspects, biologic therapies should be considered for patients with persistent SIJ pain despite steroid injections, or for those with a high inflammatory burden, as indicated by elevated CRP levels or positive MRI findings. Current recommendations suggest that biologic therapies may be particularly beneficial for patients with systemic involvement or those who have failed multiple courses of steroid injections. However, further research is required to confirm their efficacy as a first-line injective therapy.

The constraints of this systematic review and meta-analysis mirror the limitations found within the literature analyzed. The studies included in the analysis exhibited significant heterogeneity, with variations in the scores employed and the durations of follow-up. This heterogeneity poses challenges in directly comparing cohort studies, even when they investigate the same treatment modalities. Specifically, the heterogeneity in follow-up periods makes it difficult to compare results across studies and assess the long-term efficacies. Moreover, the limited number of studies further compounds these difficulties. The reporting of outcomes based on specific subtypes of SpA was lacking, and distinctions among different types of SpA were not made. While the involvement of the SIJ is crucial for SpA diagnosis and is almost universally present, the extent and systemic spread of the disease can influence systemic therapy, thereby impacting VAS and BASDAI scores. The specific details regarding additional and concomitant treatment were insufficiently described, and the wide range of treatments made it impractical to categorize patients into different groups for evaluation. The comparison of anti-TNF injections to steroid injections was hindered by the limited number of patients included in the former group. Notwithstanding the limitations mentioned, this systematic review and meta-analysis provided valuable insights into the benefits and drawbacks of the examined injective approaches in patients with SpA. The findings of this study underline the evidence and significance of various injection therapies, offering valuable clinical implications for physicians managing SIJ patients affected by SpA. Additionally, these results can guide researchers in writing new recommendations and in designing future studies aimed at optimizing SIJ treatment in SpA patients.

## 5. Conclusions

Corticosteroids have emerged as the predominant and most documented injective approach for managing SIJ pain in SpA. Studies conducted in recent decades have reported both the safety and effectiveness of corticosteroid injections. However, while corticosteroid injections have been shown to be generally safe, not all patients respond to treatment, and a significant reduction in pain relief has been documented over time. On the other hand, the current body of literature lacks consistency in supporting the efficacy of alternative injective therapies. Thus, additional studies are warranted to improve the understanding of the effectiveness of steroid therapy, identify the most optimal steroid formulation, and provide further evidence for the results of novel injective approaches to address SIJ pain in SpA patients.

## Figures and Tables

**Table 1:** Details of included studies. N.R. = non-reported; VAS = visual analog scale; BASDAI = Bath Ankylosing Spondylitis Disease Activity Index; ASDAS = Ankylosing Spondylitis Disease Activity Score; FTF = fingertip-to-floor distance; TAC = triamcinolone acetate.

First Author	Year	Study Design	N° Initial Patients (Treated)	Type of Treatment	Injected Amount	Final Follow-up	Scores	N° Failures (Definition)	N° Complications
Al Khayyat S.G. [5]	2022	Prospective case series	26	Steroid (TAC)	1 mL	6 m	VAS	0	0
Bollow M. [6]	1996	Prospective case series	66	Steroid (TAC)	1 mL	1 w	VAS	5 (not effective)	0
Braun J. [7]	1996	Prospective case series	30	Steroid (TAC)	1 mL	18 m	VAS	2 (not effective)	0
Sadreddini S. [8]	2009	Prospective case series	29	Steroid (TAC)	2 mL	5 m	VAS, 4-level likerty pain score, provocative tests	0	0
Cui Y. [9]	2010	Prospective case series	16	Biologic (etanercept)	2 mL	3 m	VAS, BASDAI	0	0
Günaydin I. [4]	2006	Prospective case series	31	Steroid (TAC)	1 mL	3 m	VAS	0	0
Hartung W. [10]	2010	Prospective case series	14	Steroid (TAC)	3 mL	1 m	VAS	3 (pain during the procedure)	0
Karabacakoglu A. [11]	2002	Prospective case series	17	Steroid (betamethasone)	N.R.	2 m	Patients’ personal satisfaction	3 (lost at follow-up)	0
Kokar S. [12]	2021	Retrospective comparative study	43	Steroid (TAC)	3 mL	1 m	VAS, BASDAI	10 (not effective)	0
Luukkainen R. [13]	1999	Prospective comparative study	20	Steroid (methylprednisone acetate)	3 mL	2 m	VAS, pain index	7 (not effective)	0
Althoff C.E. [14]	2015	Prospective comparative study	29	Steroid (TAC)	2 mL	6 m	VAS, BASDAI	6 (need steroid injection)	0
Maugars Y. [15]	1996	Randomized controlled study	10	Steroid (cortivazol)	1.5 mL	6 m	VAS, NSAID intake variation, limping evolution, unipodal jump evolution, sacroiliac	4 (not effective)	3
Maugars Y. [16]	1992	Prospective case series	24	Steroid (cortivazol)	1.5 mL	2 m	IMPROVEMENT (very good–good–fair–failure)	2 (not effective)	0
Pereira P.L. [17]	2000	Prospective case series	10	Steroid (TAC)	1 mL	3 m	VAS	0	0
Rajaei E. [1]	2022	Randomized controlled study	60	Steroid (methylprednisolone)	1 mL	2 m	VAS, BASDAI, FTF	0	0
Ramírez H.M.A. [18]	2022	Prospective case series	32	Steroid (bethametasone)	3 mL	6 m	VAS, BASDAI, ASDAS	0	0
Soliman E. [19]	2015	Prospective case series	37	Biologic (infliximab)	2 mL	6 m	VAS	0	0

## Data Availability

This study is a systematic review and meta-analysis that utilized data extracted from previously published studies. All data analyzed in this study are available in the original publications cited in the manuscript.

## Abbreviations

The following abbreviations are used in this manuscript:

N.R. = non-reported; VAS = visual analog scale; BASDAI = Bath Ankylosing Spondylitis Disease Activity Index; ASDAS = Ankylosing Spondylitis Disease Activity Score; FTF = fingertip-to-floor distance; TAC = triamcinolone acetate; SIJ = sacroiliac joint; SpA = spondyloarthropathy; US = ultrasound; TNF = anti-tumor necrosis factor; AS = axial spondyloarthritis; NSAIDs = non-steroidal anti-inflammatory drugs; CRP = C-reactive protein; DMARDs = disease-modifying anti-rheumatic drugs
